# Design and Implementation of a Stereo Vision System on an Innovative 6DOF Single-Edge Machining Device for Tool Tip Localization and Path Correction †

**DOI:** 10.3390/s18093132

**Published:** 2018-09-17

**Authors:** Luis López-Estrada, Marcelo Fajardo-Pruna, Lidia Sánchez-González, Hilde Pérez, Laura Fernández-Robles, Antonio Vizán

**Affiliations:** 1 Department of Mechanical Engineering, Universidad Politécnica de Madrid, 28006 Madrid, Spain; marcelo.fajardo.pruna@alumnos.upm.es (M.F.-P.); avizan@etsii.upm.es (A.V.); 2 Department of Mechanical, Computer and Aerospace Engineering, Universidad de León, 24071 León, Spain; lidia.sanchez@unileon.es (L.S.-G.); hilde.perez@unileon.es (H.P.)

**Keywords:** single-edge cutting, micro machining, meso machining, machine-tool, stereo vision system, tool path correction, tool tip localization

## Abstract

In the current meso cutting technology industry, the demand for more advanced, accurate and cheaper devices capable of creating a wide range surfaces and geometries is rising. To fulfill this demand, an alternative single point cutting device with 6 degrees of freedom (6DOF) was developed. Its main advantage compared to milling has been the need for simpler cutting tools that require an easier development. To obtain accurate and precise geometries, the tool tip must be monitored to compensate its position and make the proper corrections on the computer numerical control (CNC). For this, a stereo vision system was carried out as a different approach to the modern available technologies in the industry. In this paper, the artificial intelligence technologies required for implementing such vision system are explored and discussed. The vision system was compared with commercial measurement software Dino Capture, and a dedicated metrological microscope system TESA V-200GL. Experimental analysis were carried out and results were measured in terms of accuracy. The proposed vision system yielded an error equal to ±3 µm in the measurement.

## 1. Introduction

When it comes to the meso cutting machining industry, milling is the preferred choice due to its wide study and exploration [1]. It is used from the manufacturing of biochips [2] to fuel cell plates [3]. However, the high operating costs and the limitations in the tool geometries and dimensions, add additional manufacturing challenges [4]. Current studies on the topic explore the improvement of the models [5], the evaluation of the surface roughness of the tool or pieces [6], and the proper selection of optimal parameters [7].

**Figure 1:**
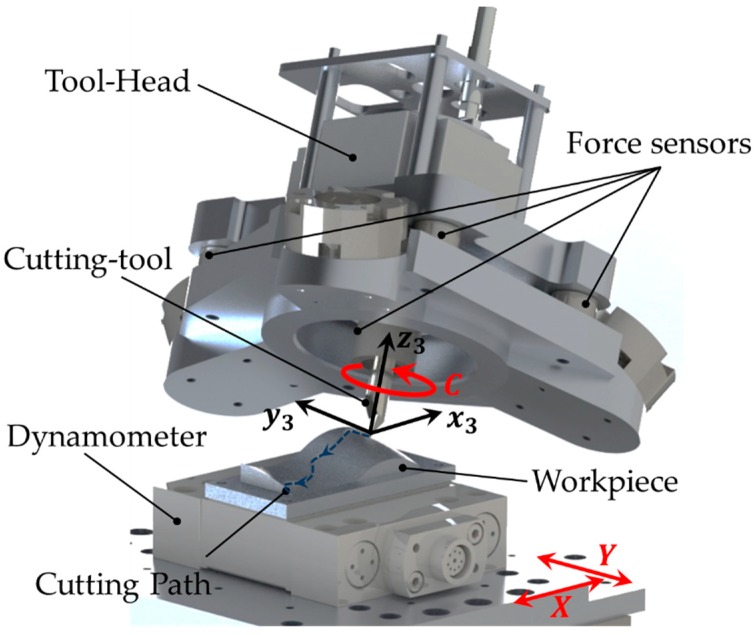
Schematic of the cutting process.

Based on cutting process modelling [8,9], a new single point cutting model has been developed. To take full advantage of this model, a 6 degrees of freedom (6DOF) machine capable of tangentially aligning the tool to the velocity path, as illustrated in Figure 1, has been developed. This system has proved to be an acceptable substitute of milling due to the competitive geometries and shapes that is able to reproduce.

One of the key components studied in the current works is to monitor the tool in order to establish when it should be replaced to keep good quality standards in the machined pieces. Some are based on indirect measurements, which model the wear of the tool by measuring other variables such as cutting forces [10], vibrations [11] and electrode’s profile [12]. Whereas other works measure the wear resistance of various tool structural parameters [13]. Other works are based on direct measures, which means that they measure the wear of the tool without using intermediate variables. New approaches use machine vision to estimate the tool wear, mainly from bottom view images of the milling tool [14,15,16]. They proved to be able to successfully locate the workpiece references [17], along with the cutting edges of the employed tools [18,19].

In contrast to tool wear monitoring, fewer amount of works in the bulk of literature focus on the correct localization of the tool tip. One of the requirements to achieve an accurate geometry of the machined pieces is to provide a good monitoring system of the device. It should be able to precisely find the tool tip. The tool tip location is measured on the axis that orientates the tool along the path. Usually, this is a relative measure to the center of the *C* axis when the rotation is produced on the *Z* axis. Most of the computer numerical control (CNC) systems, such as FAGOR 8070 used in this paper, calculate the distance with respect to the central axis. Frequently, due to the nature of the manufacturing processes and the tool wear, the tool tip is not aligned to the central axis. Therefore, a correction of the distance estimation needs to be considered in order to produce reliable measures. Pérez et al. [20] used cutting forces to estimate the tip localization. Xuewei et al. [21] made use of the tool deflections to correct the tip path, whereas Singh and Dvivedi [22], and Yu et al. [23] used machine vision techniques with the same purpose.

To locate the tool tip on the 6DOF device, different technologies were analyzed, each varying in accuracy and costs. Given the dimensional restrictions of the device, and the required budget, the system must be compact, modular and low cost. With this in mind, a vision system implemented in a Personal Computer (PC) was preferred [24]. The flexibility, low costs and expandability are desired characteristics of such systems to obtain the pursued results.

## 2. Description of the 6DOF Single-Edge Machining Device

**Figure 2:**
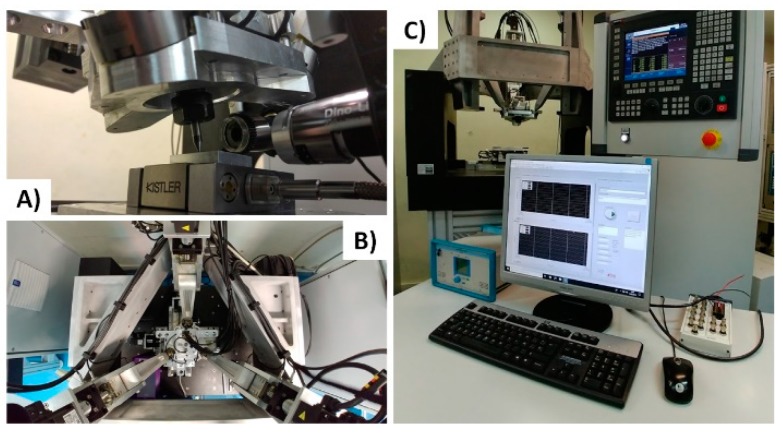
Configuration of the 6DOF hybrid single-edge cutting device. (**A**) Tool head with dual camera vision system and single-edge tool, (**B**) parallel mechanical configuration, (**C**) CNC control and PC for the process monitoring and analysis.

For the proposed single-edge cutting process, it is crucial to obtain a user-friendly system with high precision. For this, a 6DOF device was designed and developed, able to fully position a single-edged tool in translations and rotations [25], Figure 2 illustrates the configuration of the 6DOF hybrid single-edge cutting device. A parallel mechanism constituted of three parallel-revolute-spherical configuration (3PRS) is used to position the tool head in the desired orientation and with a XY-Stage compensates for the parasitic movements of the tool, a *C* axis rotates the tool on its longitudinal axis to maintain the orientation of the cutting edge at the same time as maximizing the working space. Then, the axes controlled by the CNC Fagor 8070© are Z1, Z2, Z3, X, Y, C. The machine tool has a force measuring system in the 3PRS tool head made up of 4 KISTLER triaxial sensors and in the XY-Stage a KISTLER 9256C1 dynamometer. All these elements in conjunction with a stereo vision system monitor the cutting process. The information obtained from the vision system is crucial to make the proper corrections on the CNC code. 

### Inverse Kinematics of the 6DOF Machine Tool

**Figure 3:**
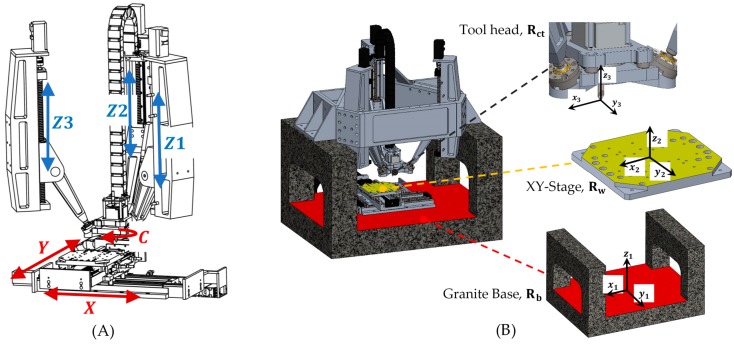
(**A**) Axis of the hybrid mechanism; (**B**) reference frames in the 6DOF machine tool.

To analyze the inverse kinematics of the 6DOF machine tool three different frames are defined. One on the base $R_{b}\left(\begin{array}{ccc} x_{1} & y_{1} & z_{1} \end{array}\right)$, other in the XY-Stage $R_{w}\left(\begin{array}{ccc} x_{2} & y_{2} & z_{2} \end{array}\right)$ and finally, in the cutting tool tip end $R_{ct}\left(\begin{array}{ccc} x_{3} & y_{3} & z_{3} \end{array}\right)$ as is shown in Figure 3. The goal of these calculations is to define the position of the tool-tip $P={\left[\begin{array}{cccccc} P_{x} & P_{y} & P_{z} & \alpha & \beta & \gamma \end{array}\right]}^{T}$ at every time during the machining process (Figure 1).

**Figure 4:**
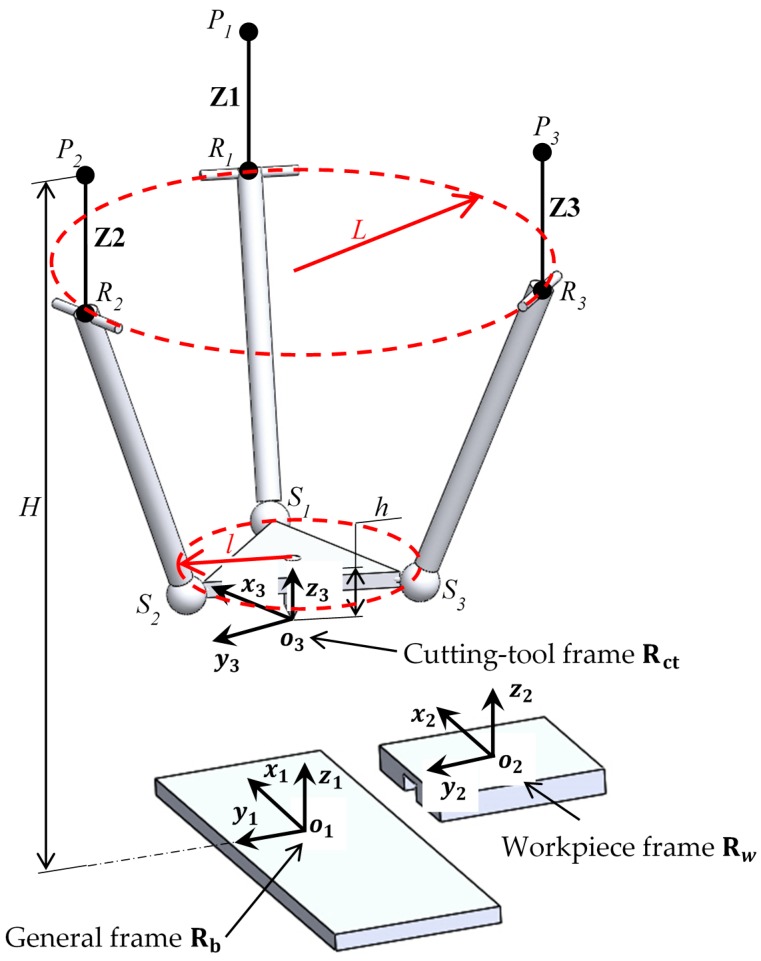
Kinematic parameters of the 3PRS+XY+C hybrid machine tool.

Since the mechanism has two main devices, a parallel mechanism and a XY-Stage, it is necessary to relate their movements using a rotation matrix, $R=R_{z_{1}x_{1}y_{1}}=R_{y_{1}\left(\beta \right)}R_{x_{1}\left(\alpha \right)}R_{z_{1}\left(\gamma \right)}$ [26]. The XY-Stage is a serial mechanism, then its movement is directly related to the actuation in X and Y axes. On the other hand, the parallel mechanism does not actuate directly over their 3DOF. The actuation is performed on the Z1, Z2 and Z3 axes (Figure 4) and results in two rotations and one translation [27]. Finally, the C axis must rotate to positioning the cutting tool tip in the trajectory and to compensate the rotational parasitic motion of the 3PRS mechanism. All computations are realized relative to the fixed frame in the granite base.

The Euler angles α and β are the rotational DOFs that relates the frame $R_{ct}$ to $R_{b}$ in the axes *x* and *y*. These movements induce a rotational parasitic motion $\gamma_{b}$ in *z* axis which is defined by Equation (1) and two additional parasitic motions in *x* (Equations (2)) and *y* (Equation (3)), corresponding to the translation of the tool tip position $o_{3}$ [28]. Where, c(∗) and s(∗) correspond to the cos(∗) and sin(∗). The Z1, Z2 and Z3 actuations to the input positions α, β and $P_{z}$ of the P cutting path are expressed by Equation (4) [29]:

(1) $$\;\gamma_{b}=\arctan\left(\frac{s\alpha s\beta }{c\alpha +c\beta }\right)\;$$

(2) $$\;o_{3_{x}}=l\left(c\beta s\gamma -s\alpha s\beta c\gamma \right)-hc\alpha s\beta \;$$

(3) $$\;o_{3_{y}}=\frac{l}{2}\left(-\sqrt{3}c\alpha s\gamma +c\alpha c\gamma -\sqrt{3}c\beta s\gamma +\sqrt{3}s\alpha s\beta c\gamma -c\beta c\gamma -s\alpha s\beta s\gamma \right)+hs\alpha \;$$

(4) $$\;Zi=H-S_{i_{z}}-\sqrt{{\bar{R_{i}S_{i}}}^{2}-\left[{\left(R_{i_{z}}-S_{i_{z}}\right)}^{2}-{\left(R_{i_{y}}-S_{i_{y}}\right)}^{2}\right]}\;\;\;\;i=1,\;2,\;3\;$$

where $S_{i}=\left[\begin{array}{ccc} S_{i_{x}} & S_{i_{y}} & S_{i_{z}} \end{array}\right]$ and $R_{i}=\left[\begin{array}{ccc} R_{i_{x}} & R_{i_{y}} & R_{i_{z}} \end{array}\right]$ are the positions vectors of the spherical and rotational joints respectively in the general reference frame $R_{b}$. The geometrical parameters to the device are shown in Table 1.

The rotation of the C axis considering the rotational parasitic motion $\gamma_{b}$ and the positioning of the cutting tool to the angle γ is obtained from Equation (5). Thus, the movements of the driven axes of the machine tool X and Y considering the translational parasitic motion and the positioning of the cutting tool in $P_{x}$ and $P_{y}$ are obtained by Equations (6) and (7). Z1, Z2, Z3 and C are expressed by Equations (4) and (5).

(5) $$\;C=\gamma -\gamma_{b}\;$$

(6) $$\;X=P_{x}+O_{3_{x}}\;$$

(7) $$\;Y=P_{y}+O_{3_{y}}\;$$

To define the actual position of the cutting edge, it is necessary to measure the deviation of the tool tip in the $R_{ct}$ frame relative to the $z_{3}$ axis. The measured values that allows to correct the tool-tip position are $\Delta_{x}$, $\Delta_{y}$ and Δθ which correspond with the offset in $x_{3}$, $y_{3}$ and the angular position around $z_{3}$ respectively.

Thus, the machine tool has the following mechanical specifications: Each linear axis, X, Y, Z1, Z2 and Z3 has a maximum feed of 10.000 mm/min and the *C* axis has maximum velocity of 120 rpm. The maximum reachable Euler angles of the tool head α and β are ±30°, the travels of the X and Y axis are 100 mm and for Z1, Z2 and Z3 are 190 mm. The stiffness of the device has an anisotropic behaviour because given the nature of the parallel mechanism, the minimum stiffnesses are $1.1\times 10^{4}$ N/mm for X and Y axis and $6.2\times 10^{4}$ N/mm. The maximum stiffnesses are $1.41\times 10^{4}$ N/mm for X and Y axis and $6.7\times 10^{4}$ N/mm. The maximum workpiece size depends of the cutting tool length with an average workpiece size of 80×80×100 mm, within this space it is possible to fully reach the full potential of the orientation and positioning of the tool. The mode shapes of the parallel mechanism are 15 between 16 and 61 Hz [30] that characterizes the main vertical, horizontal and rotational motions.

**Figure 5:**
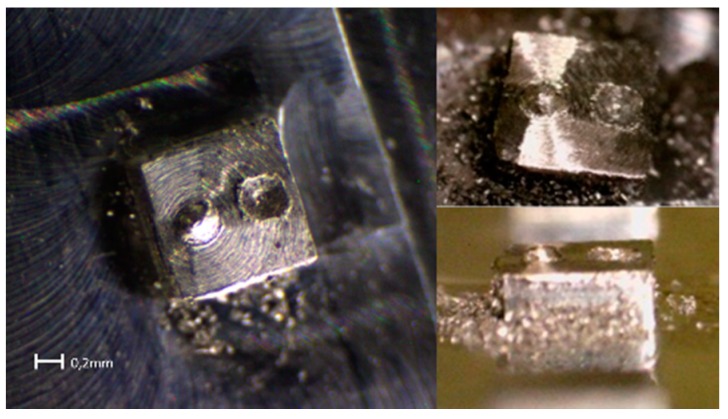
Examples of machined pieces obtained in geometric tests.

A set of preliminary tests were performed in order to evaluate the precision of the machine. The device, made of parts, worked correctly. Very different geometries were obtained, and the integration of the ISO code in CAD/CAM environments by means of the FAGOR CNC controller alleviated the translation of the CAD drawings into machining paths. Figure 5 presents some examples of the machined pieces after performing preliminary geometric tests.

## 3. Stereo Vision System

### 3.1. Materials

**Figure 6:**
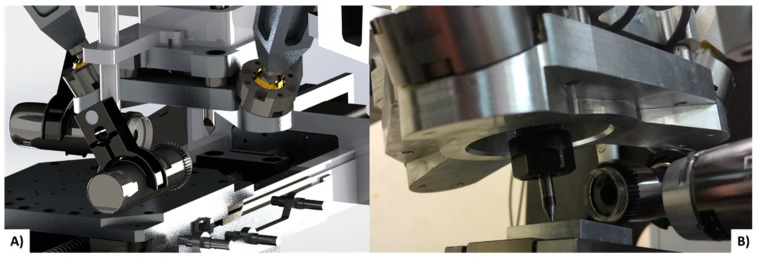
Configuration of the vision system, the cameras are placed in a perpendicular position aligned to *X* and *Y* axis, (**A**) 3D CAD model of the designed stereo vision system on the machine; (**B**) real stereo vision system set up on the machine.

The hardware for the stereo vision system was selected based on the principles of such configurations [31]. A stereo camera configuration was chosen as an array of two DinoLite AM7515MZT cameras with an optical zoom of 273×, a resolution of 5 Megapixels and a 1.3 ratio. The lenses of these cameras were designed for measuring purposes and are able to obtain clear un-deformed images. DinoCapture is software that comes with the cameras and is able to measure geometries in images after a manual procedure. Our own vision system was designed by means of the vision toolbox of Matlab. The dimensions of the device allow configuring a diverse range of arrangements of the cameras depending on the geometries that need to be machined and the accessories used. The cameras need to be placed in a perpendicular position, one in the plane XZ and the other in the plane YZ. Figure 6 illustrates the designed CAD vision system and the real vision system that was implemented on the device. As a highlight, the design of the vision system is modular, which allows making different arrangements of the elements in the device. Integrated LEDs are provided with the cameras, which are used for illumination purposes. Nevertheless, if the conditions of the machining or the materials of the pieces need a different illumination, other light sources can be added to the device due to its modularity.

### 3.2. Schema of the Stereo Vision System

**Figure 7:**
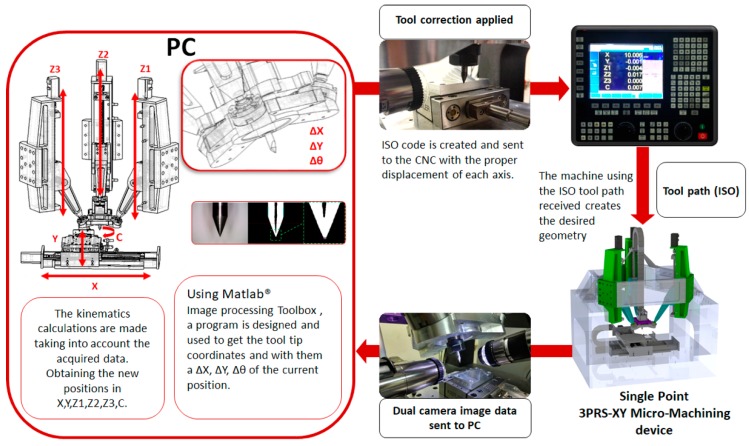
Schema of the image capturing process and the tool path corrections.

**Figure 8:**
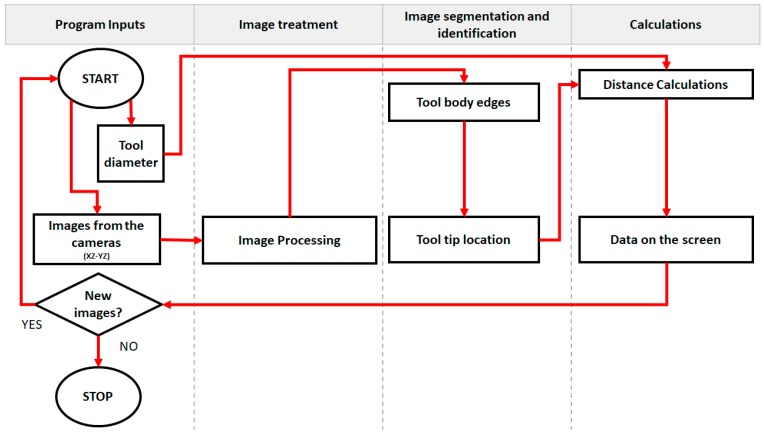
Basic flow diagram of the stereo vision method.

The stereo vision system was mounted on the device as an accessory, it is integrated on the 6DOF tool head and can be removed or expanded depending on the process. The image processing and the computations of the vision system for tool tip localization and path correction were calculated using Matlab on a PC. Once the computations are made, the information is sent to the FAGOR 8070 CNC controller, and with the delta obtained from the offset of the tool tip, a correction is calculated and applied to the tool path. This process is repeated so the stereo vision system is able to track the tool tip, then the bed can be moved away. Figure 7 shows an illustration about the image capturing of the tool tip, get the position of the tool tip, compute the error and correct it and displace the tool properly. Figure 8 shows the general flow diagram of the stereo vision system, which consists of 4 basic stages, program Inputs, image treatment, tool edge and tip localization, and calculations. The program inputs stage consists on gathering data such as the tool diameter and the images captured by the cameras. The image treatment stage transforms the captured images to graphic processing unit (GPU) arrays to take advantage of the 3D acceleration card of the PC, then the images histogram is normalized and turned into gray scale images, a Gaussian filter is applied and then turned into negative images (color inverted), isolated pixels are filled, and the images are turned into binary arrays. On the tool edge and tip localization stage the algorithm scans both sides of the tool to find its edges (using a technique based on canny edge detection), a calculation is made to draw and extend the central axis of the tool, with this data the algorithm scans for a convergence on the tool tip, the obtained coordinates are stored. The final calculation stage consists of the use of the data obtained in previous stages to calculate the relation of millimeters per pixel and by orthogonal projection of the coordinates of the tool tip the distance of the tool tip relative to the axis of tool is obtained, then the data is presented on the PC monitor overlayered on the original images obtained by the cameras.

#### 3.2.1. Image Acquisition

**Figure 9:**
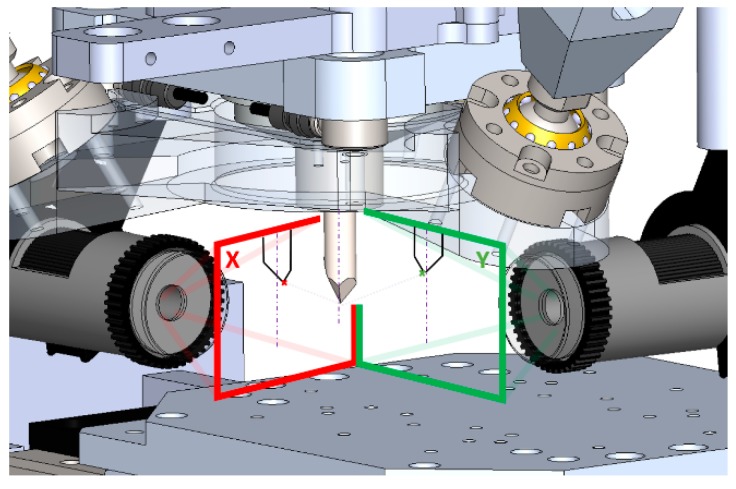
Illustration of the computation of the tool tip using stereo vision.

The acquired color pictures were captured at a resolution of 1280 × 960 pixels, following a dual camera arrangement based on stereo vision systems [32]. A schematic of the estimation of the tool tip using the stereo vision system is shown in Figure 9. Such systems are very practical for precisely finding the location of a point in a three-dimensional space using a triangulation calculation, after an appropriate calibration of the cameras, which is critical [33]. The location of the tool tip was computed in this way. The diameter and axis of the tool were considered as reference point for both cameras. This affects directly the accuracy of the vision system.

#### 3.2.2. Tool Localization

Both cameras take an image of the tool on the planes XZ and YX (which represents the $x_{3}z_{3}$ and $y_{3}z_{3}$ of the machine), the images must be properly processed to obtain the information that is required. An intelligent processing of the visual information based on vision sensors system is applied. Matlab image processing toolbox was chosen due to the variety of image processing methods implemented and the flexibility of its programming language when creating new algorithms [34]. The selection of these techniques will have a great impact on the accuracy of the obtained dimensions [35]. The images are preprocessed by first converting them into grayscale and then normalizing them to stretch the contrast of the images. This reduces the errors that can be produced by the light source (in this case the integrated LEDs of the DinoLite cameras), which have a direct effect on the segmentation of the image. A poor segmentation will result in a bad identification of the object, that is, the cutting tool. Finally, the image is color inverted, black to white and vice versa.

Then, the inverse image is thresholded [36] and converted into a binary image in which a logical one indicates a pixel that belongs to the tool whereas a logical zero indicates otherwise. The tool body is metallic and cylindrical, which can cause reflections on the tool surface. This issue may lead to defaults in the binarizing process, and thus, on the tool tip localization. In this case, isolated black pixels that actually belonged to the tool were found. Gaussian filters and filling tools were used to alleviate this undesired effect. Even though the reflections vary from images and poses, the conceived algorithm became robust against reflections.

#### 3.2.3. Alignment of the Tool with Respect to the Cameras

**Figure 10:**
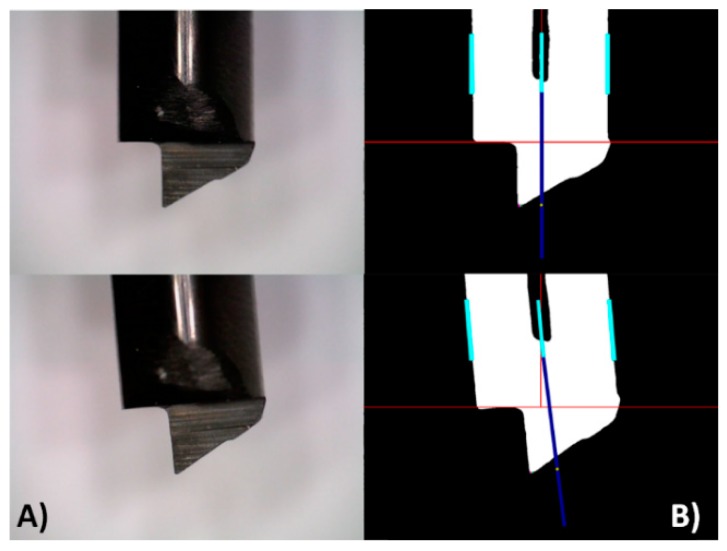
Two examples of the tool alignment process. (**A**) Images without processing, showing a good alignment and a bad alignment; (**B**) image processed also in both cases, the cyan segments represent the two edges of the tool body. The dark blue line represents the lengthen parallel mid-segment. In addition, the red lines represent the extra guide lines.

Once the tool is located, the next step is to localize the central axis of the tool body. The two long edges of the tool body are found and defined with two segments or vectors. Then, the parallel vector placed in the middle position of the two is geometrically interpolated. Extra guide lines are calculated to ease the alignment of the camera in relation to the pose of the tool body, as shown in Figure 10. Special mechanical supports were designed so the guide lines are also aligned to the device, and with this, two Regions of Interest (ROI) are defined [37], the tool body and the tool tip.

The two edges of the tool body and the real dimension in millimeters of the tool diameter are used to estimate the pixels per millimeter ratio. The parallel mid-segment is lengthened to a straight line to better visualize the central axis of the tool body. The tool tip localization will focus on the area surrounding the automatically located mid-line of the tool body.

#### 3.2.4. Tool Tip Localization

The first step of the tool tip localization consists of acquiring the tool diameter as an input variable. The algorithm is designed to find the tool body. Again, as the device only admits cylindrical tool body shapes, this will be the reference point for the subsequent calculations.

**Figure 11:**
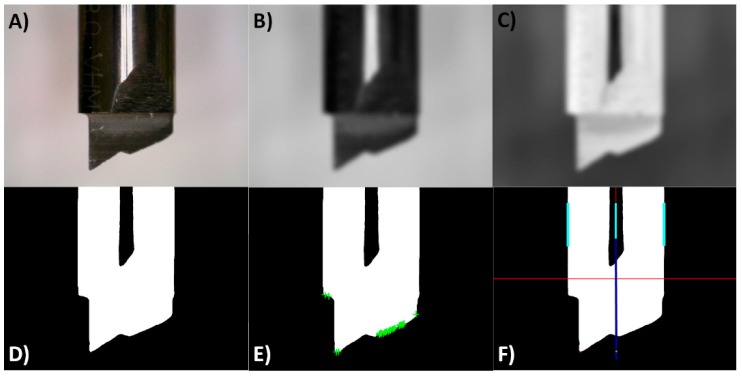
Different stages of the image processing: (**A**) the original image is captured; (**B**) histogram normalization, gray scaling and Gaussian filter is applied; (**C**) inverse image is obtained; (**D**) cleaning of the image and object detection; (**E**) edge localization; (**F**) tool alignment and calculations to find the tool tip.

Once the tool body is identified, the next step is the localization of the tool tip. The tool tip can be considered as a corner in image processing. Harris and Stephens [38] algorithm was used to find the tool tip. This method combines corner and edge detectors based on the local auto-correlation function, and it is applied to the binary image. Figure 11 shows different snapshots of different stages of the image processing and tool tip localization.

Once the tool tip is located, the coordinates of the tool tip position are kept in two different variables, one for each camera. These coordinates on the image are measured in pixels. Then, the coordinates of the tool tip are projected on the reference mid-line. Therefore, the distance between the tool tip and the central axis of the tool body can be measured in pixels. Finally, the pixel measurements are transformed into millimeters using the previously obtained ratio.

#### 3.2.5. Path Correction

**Figure 12:**
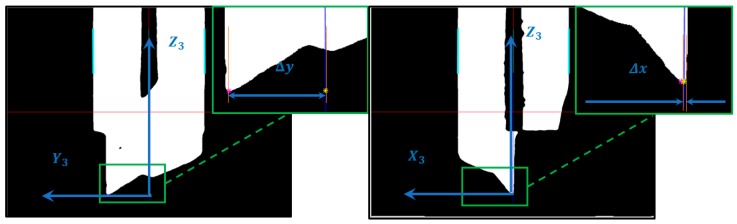
Example of a tool tip with offset relative to the reference frame of the device (**Δ*_x_***, **Δ*_y_***).

By default, the configuration of the machine tool considers as initial position of the tool tip on the origin of a coordinate system $R_{ct}$. However, given the variety of the single edge tool geometries, the real position of the tool tip is not aligned to this reference frame, as shown in Figure 12, and can vary significantly.

**Figure 13:**
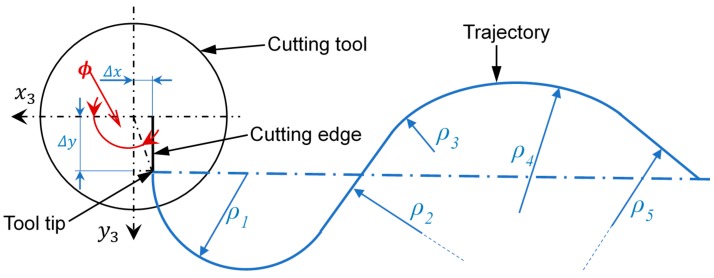
Tool path and tool rotation schematic.

To make the proper corrections on each of the axis of the device it is necessary to measure the relative deviation to the tool planes $R_{ct}$ in an initial position on the C axis. These values are determined as $\Delta_{x}$, $\Delta_{y}$ and Δθ, Equation (8), and are represented in Figure 13. To keep the cutting edge tangent to the trajectory P during the cutting process, it is necessary to correct the displacements generated by the movement of the tool tip when the C axis rotates according to Equations (11) and (12). These displacements correspond to the movements of a circle of radius $r_{\Delta }$, the real position of the cutting edge ϕ, where γ is the sum of the angular parasitic motion of the 3PRS mechanism and the actuation of the C axis, Equation (9).

(8) $$\;\Delta \theta =\mathrm{atan}\left(\frac{\Delta_{x}}{\Delta_{y}}\right).\;$$

(9) $$\;\phi =\frac{\pi }{2}+\Delta \theta +\gamma \;$$

(10) $$\;r_{\Delta }=\sqrt{\Delta_{x}^{2}+\Delta_{y}^{2}}\;$$

(11) $$\;\delta_{\mathrm{xr}}=r_{\Delta }\cos\left(\phi \right)\;$$

(12) $$\;\delta_{\mathrm{yr}}=r_{\Delta }\sin\left(\phi \right)\;$$

Applying the rotation matrix $R=R_{z_{1}x_{1}y_{1}}=R_{y_{1}\left(\beta \right)}R_{x_{1}\left(\alpha \right)}R_{z_{1}\left(\gamma \right)}$ a relation between the orientation of the tool and the general coordinate reference system is obtained [39], and with it the values of the correction on the work planes of the device $x_{1}y_{1}z_{1}$, Equation (13).

(13) $$\;{\left[\begin{array}{c} \delta_{x}\\ \delta_{y}\\ \delta_{z} \end{array}\right]}_{R_{b}}=R{\left[\begin{array}{c} \delta_{xr}\\ \delta_{yr}\\ 0 \end{array}\right]}_{R_{ct}}\;$$

As a final step, the obtained values act as a correction of the theoretical values from the programmed toolpath for each of the axis of the device [28] with Equations (14)–(16). Where X, Y and Zi are the actuations when $\Delta_{x}$ and $\Delta_{y}$ are 0 mm from Equations (4), (6) and (7).

(14) $$\;X_{p}=X+\delta_{x}\;$$

(15) $$\;Y_{p}=Y+\delta_{y}\;$$

(16) $$\;Zi_{p}=Zi+\delta_{z}\;$$

## 4. Results

In this section, the validation of the vision system performance and its efficiency is presented. For this, the results yielded with the proposed system are related with two different measuring systems: TESA V-200GL and DinoCapture.

TESA V-200GL is a dedicated metrological microscope system. This system provides the coordinates with respect to the central axis of the tool body. The displacement in x axis relative to the measuring system indicates the distance to the tool tip, YZ = 3.016 mm and XZ = 0.017 mm. These measurements are the base line to compare the capabilities of the vision system.

**Figure 14:**
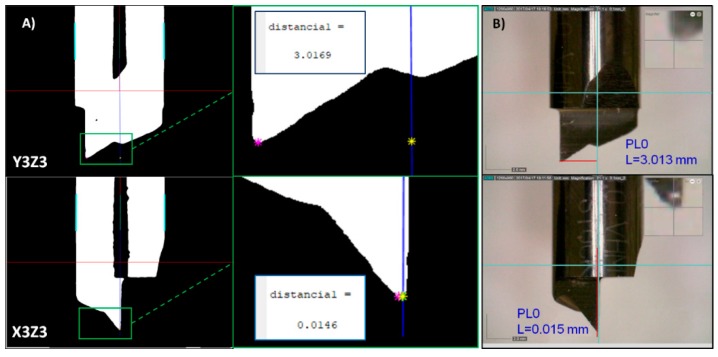
Measure of the tool tip with respect to the central axis of the tool body on the coordinated system $x_{3}y_{3}z_{3}$. (**A**) The image shows the results achieved with the proposed stereo vision system; (**B**) the image shows the results achieved by DinoCapture software.

Forty-five images per tool plane, YZ and XZ which represent in the machine the $y_{3}z_{3}$ and $x_{3}z_{3}$ planes, were taken. The proposed method was used to automatically obtain the tool tip measures with respect to the central axis of the tool body. The same measurement was obtained with DinoCapture, the software included with the cameras. Two examples of such comparisons are shown in Figure 14.

The designed stereo vision system obtained $y_{3}z_{3}$ = 3.0169 mm and $x_{3}z_{3}$ = 0.0146 mm, while the DinoCapture software achieved $y_{3}z_{3}$ = 3.013 mm and $x_{3}z_{3}$ = 0.015 mm. Thus, both systems obtained similar results. Mind that the results achieved with the TESAV-200GL were considered as the base line. The differences between the base line measurement and the measurement from the vision system are gathered in Table 2. The errors yielded by the proposed stereo vision system are lower when the tool tip is located further away to the tool axis $y_{3}z_{3}$ and $x_{3}z_{3}$, whereas the are significantly higher when it is placed close to the $x_{3}z_{3}$ axis. Nevertheless, the accuracy of the proposed system is acceptable for any position of the tool tip.

The range of error for the proposed stereo vision system and the DinoCapture software was around ±3 µm. However, DinoCapture software has a great drawback with respect to the proposed system, it needs to be manually calibrated through point and click maneuvers, whereas the proposed system is fully automatic.

**Figure 15:**
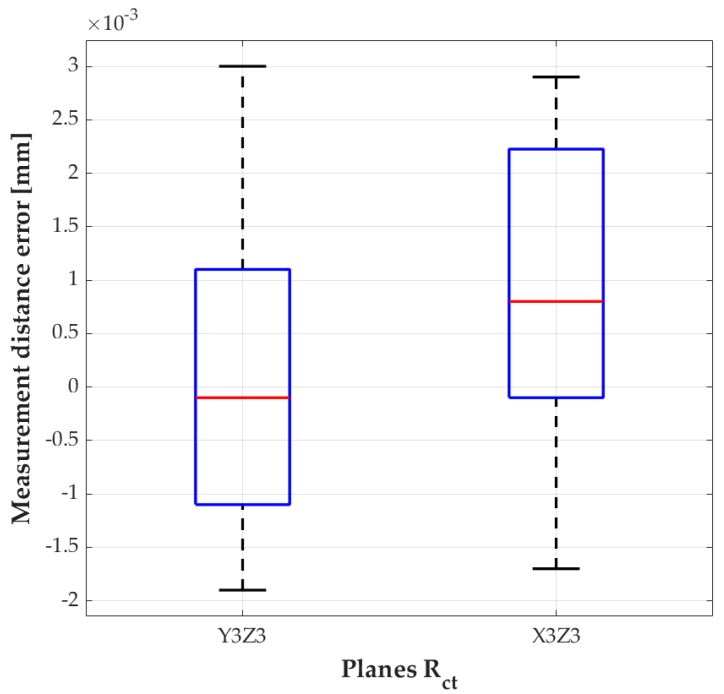
Boxplot of the vision system measurements, YZ camera and XZ Camera.

Figure 15 shows box plots for the measurements of the images captured by the vision system, and it is noticeable that the results were achieved with high confidence rate. The error is produced by lighting conditions, in this case, the integrated camera LEDs (4000 lumens in the tool tip area).

**Figure 16:**
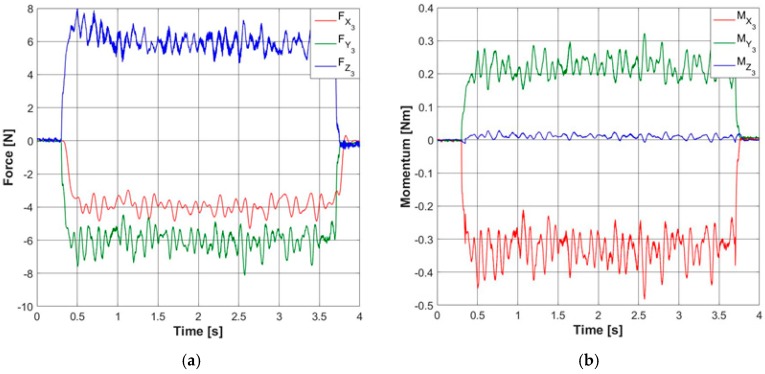
(**a**) Forces in the cutting process of the linear path ($F_{c}=F_{y_{3}}$, $F_{f}=F_{x_{3}}$, $F_{t}=F_{z_{3}}$); (**b**) momentums of the linear path.

Since any deviation of the tool tip will give as a result a variation on forces and momentums on the tool head of the device, four triaxial sensors have been used on the tool head to make an indirect validation. A linear cutting path using the measured tool and a depth of cut of 0.04 mm is made. The forces and momentums of the process are obtained, as shown in Figure 16.

**Figure 17:**
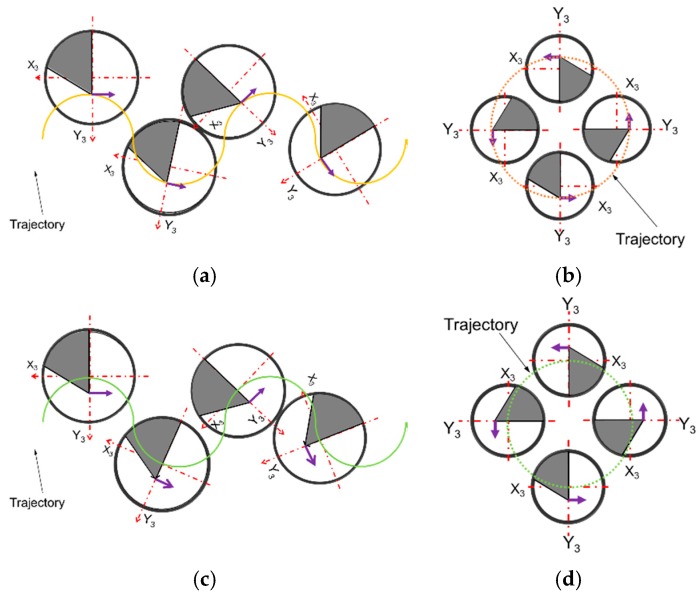
Test paths, (**a**) Spline path using the correction calculations; (**b**) circular path using the correction calculations; (**c**) spline path without the correction calculations; (**d**) circular path without correction calculations.

Using the position of the tool tip in a vector $R=\left[\begin{array}{ccc} -0.015 & 3.013 & -60 \end{array}\right]\;\mathrm{mm}$ and using the mean values of the forces captured by the sensors $F=\left[\begin{array}{ccc} -3.97 & -6.00 & 5.92 \end{array}\right]\;N$, the corresponding momentum (M=R×F) is calculated $M=\left[\begin{array}{ccc} -0.3422 & 0.2383 & 0.0121 \end{array}\right]\;\mathrm{Nm}$. This calculation is compared with the mean value obtained from the momentum sensors $M=\left[\begin{array}{ccc} -0.3278 & 0.2241 & 0.0113 \end{array}\right]\;\mathrm{Nm}$. As it can be seen, this is a good indication of the reliability of the stereo vision system for tool tip localization and proves that the device correctly operates with reliable accuracy. To validate the tool path correction, two paths are programmed and tested in the CNC, as shown in Figure 17. The paths consist of a circle of different radius and a spline with two different radii, using both, the tool correction and without it. The obtained paths are measured and compared with the ones programmed.

**Figure 18:**
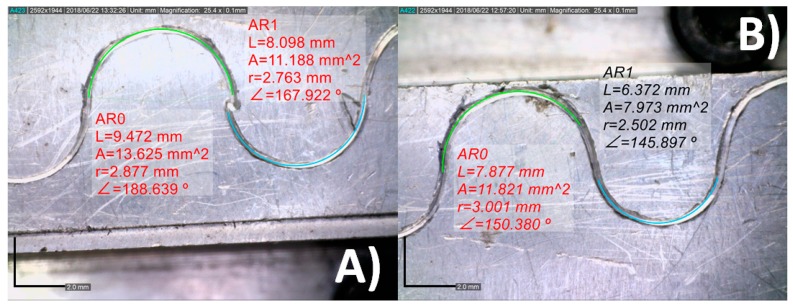
Comparison of the paths obtained, (**A**) spline path without correction; (**B**) spline path with the correction.

The tool tip is obtained with the vision system, a $\Delta_{x}\;$= 0.0188 mm and a $\Delta_{y}=1.2361\;\mathrm{mm}$ are obtained. Using this data, a spline is programmed with an arch radius of 3 mm followed by a 2.5 mm radius arch, the path is machined on a test workpiece of Aluminium 7075-T6 and using a tungsten single-edge cutting tool in an orthogonal configuration. The path without correction (Figure 18A) shows a clear deviation in the target measure of both arches. It can also be noticed a defect where the two arches link, this is due to the interpolation of the CNC to match the desired path. However, since the tip of the tool is not aligned with its central axis, the tool tip cuts along a non-desired place. The path with the correction enabled (Figure 18B) has a high accuracy following the programmed path, and it does not have the defect observed on the path without correction. The programs for the cutting paths of Figure 18 are shown in Appendix A.1.

**Figure 19:**
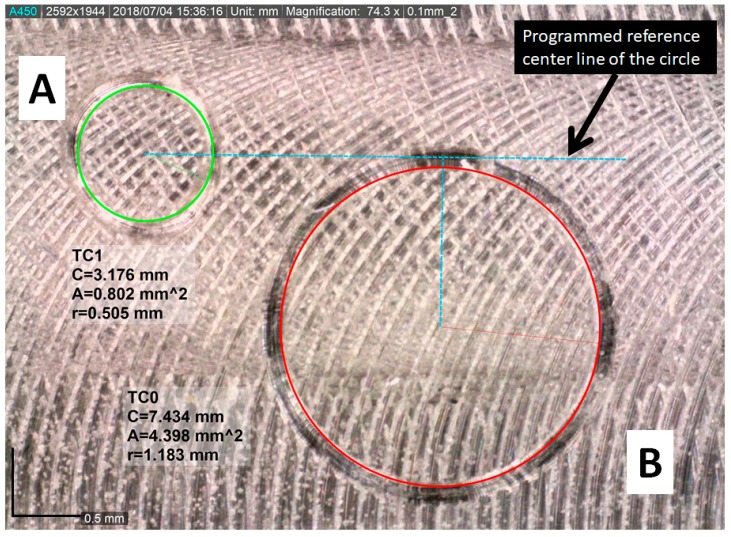
Comparison of the obtained circular paths, (**A**) circular path with correction; (**B**) circular path without correction.

The circular paths show a similar behavior, the error between the programmed path and the real one is notorious. In Figure 19, a circular path of 0.5 mm of radius is programmed, circle A uses the correction of the tool tip, circle B uses no correction. Whereas the programmed path is the same, overlaid with a blue dashed line in Figure 17, the error of positioning given the default point of reference of the axis of the tool creates an offset on dimensions and positioning. The programs for the cutting paths of Figure 19 are shown in Appendix A.2.

## 5. Discussion

In this work, a stereo vision system was designed and programmed to precisely localize the tool tip and reliably estimate the path corrections for a 6DOF single point machining device. Experimental results proved that the proposed system fulfills the accuracy range of both standard software to measure geometries provided by market cameras, DinoCapture, and dedicated metrological microscope systems, TESA V-200GL. Vision systems based on artificial intelligence are becoming a crucial application to monitor, inspect and control machines and systems in the industry. Many applications benefit from machine vision systems, which recently became low-cost and provide fast computational capacities [40]. Vision systems in micro and meso machining processes faces a promising future, the creation of both sensors and lenses capable of delivering a clear undeformed image is essential to achieve precise and accurate measurements and results. In future works, other light sources will be evaluated; given the reflective nature of metals, the contour of the tool can be affected if the light conditions are inadequate [41].

## 6. Conclusions

The proposed stereo vision system is an effective strategy for the localization of the tool tip in the machining processes. The error was delimited in a range of ±3 µm, which is comparable to commercial software and dedicated metrological microscope systems but makes up a completely automatic solution. The integrated LEDs on the cameras were used as light sources to illuminate the metallic tool. Even though results were satisfactory, other light sources can be studied in future works. Also, in future works, reference images of the tool bodies can be taken and the calculation of measurements can be tested by means of a cascade algorithm. The implementation in a 6DOF single-edge cutting machine was a success, showing that the tool tip path correction is viable in such paths. The system can create accurate cutting paths, which leads to more reliable cutting geometries.

## Figures and Tables

**Table 1:** Geometrical parameters of the 3PRS+XY+C machine tool.

Parameter	Description	Value
L	Radius of the fixed base	180 mm
l	Radius of the tool head	80 mm
H	Height of the start of Zi axes	630 mm
$\bar{R_{i}S_{i}}$	Rod length	200 mm
h	Cutting tool length	60 mm

**Table 2:** Distance errors (mm) for the acquired images, YZ camera ($y_{3}z_{3}$) and XZ camera ($x_{3}z_{3}$).

Image	$\;y_{3}z_{3}\;$	$\;x_{3}z_{3}\;$	Image	$\;y_{3}z_{3}\;$	$\;x_{3}z_{3}\;$	Image	$\;y_{3}z_{3}\;$	$\;x_{3}z_{3}\;$
1	0.0010	−0.0008	16	−0.0013	0.0028	31	0.0019	0.0024
2	−0.0007	0.0001	17	0.0002	0.0006	32	0.0005	0.0005
3	−0.0011	−0.0016	18	−0.0016	0.0020	33	−0.0015	−0.0013
4	−0.0009	0.0001	19	−0.0005	0.0024	34	0.0001	−0.0014
5	0.0014	0.0029	20	0.0001	0.0020	35	−0.0012	0.0016
6	0.0003	0.0024	21	−0.0011	0.0023	36	−0.0007	0.0020
7	0.0026	−0.0013	22	−0.0014	0.0021	37	0.0001	0.0002
8	0.0024	0.0006	23	−0.0019	0.0028	38	0.0018	−0.0002
9	0.0023	−0.0012	24	0.0030	−0.0002	39	−0.0003	0.0009
10	−0.0004	0.0020	25	−0.0013	0.0016	40	0.0026	0.0020
11	0.0005	0.0002	26	−0.0001	0.0003	41	−0.0001	−0.0017
12	0.0021	−0.0001	27	−0.0019	−0.0005	42	−0.0003	0.0026
13	0.0005	0.0028	28	0.0004	0.0005	43	−0.0006	0.0025
14	0.0023	−0.0001	29	0.0006	0.0003	44	−0.0015	0.0023
15	0.0027	0.0012	30	−0.0010	0.0008	45	−0.0019	0.0022

## Appendix A

### Appendix A.1. ISO Programs for the Cutting Paths of Figure 17

#### Appendix A.1.1. ISO Program without Tool Tip Correction

V.A.ORGT[2].X = −71.676
V.A.ORGT[2].Y = 21.309
V.A.ORGT[2].Z1 = −140.630
V.A.ORGT[2].Z2 = −140.630
V.A.ORGT[2].Z3 = −140.630
V.A.ORGT[2].C = 80
G55
P1 = 0
G01 X0 Y0 Z1 = 0 Z2 = 0 Z3 = 0 C0 F100 (PRIORITY 1 BEGIN)
$WHILE P1 ≤ 44
P1 = P1 + 11
G17 G71 G94
G02 X3 Y3 I3 J0 C90
G02 X6 Y0 I0 J3 C180
G03 X8.5 Y-3 I-3 J0 C90
G03 X11 Y0 I0 J-2 C180
G158 XP1
$ENDWHILE
G01 Z1 = 1 Z2 = 1 Z3 = 1 F50
G01 X-55 F500
G54
G158
M3

#### Appendix A.1.2. ISO Program with Tool Tip Correction

V.A.ORGT[2].X = −71.676	N220 G01 X-0.300 Y-1.027 C21.0	N3320 G01 X10.378 Y1.330 C29.0
	N230 G01 X-0.305 Y-1.078 C22.0	N3330 G01 X10.424 Y1.294 C28.0
V.A.ORGT[2].Y = 21.309	N240 G01 X-0.309 Y-1.129 C23.0	N3340 G01 X10.470 Y1.257 C27.0
V.A.ORGT[2].Z1 = −140.630	N250 G01 X-0.312 Y-1.180 C24.0	N3350 G01 X10.514 Y1.219 C26.0
V.A.ORGT[2].Z2 = −140.630	N260 G01 X-0.314 Y-1.232 C25.0	N3360 G01 X10.558 Y1.181 C25.0
V.A.ORGT[2].Z3 = −140.630	N270 G01 X-0.315 Y-1.283 C26.0	N3370 G01 X10.602 Y1.141 C24.0
V.A.ORGT[2].C = 80	N280 G01 X-0.315 Y-1.334 C27.0	N3380 G01 X10.644 Y1.101 C23.0
	N290 G01 X-0.315 Y-1.385 C28.0	N3390 G01 X10.686 Y1.060 C22.0
G55	N300 G01 X-0.313 Y-1.436 C29.0	N3400 G01 X10.728 Y1.019 C21.0
P1 = 0	N310 G01 X-0.311 Y-1.488 C30.0	N3410 G01 X10.768 Y0.976 C20.0
	N320 G01 X-0.308 Y-1.539 C31.0	N3420 G01 X10.808 Y0.933 C19.0
G01 X0 Y0 Z1 = 0 Z2 = 0 Z3 = 0 C0 F100 (PRIORITY 1 BEGIN)	N330 G01 X-0.303 Y-1.590 C32.0	N3430 G01 X10.847 Y0.890 C18.0
	N340 G01 X-0.298 Y-1.641 C33.0	N3440 G01 X10.885 Y0.845 C17.0
	N350 G01 X-0.292 Y-1.692 C34.0	N3450 G01 X10.923 Y0.800 C16.0
$WHILE P1 <= 44	N360 G01 X-0.285 Y-1.743 C35.0	N3460 G01 X10.959 Y0.755 C15.0
	N370 G01 X-0.278 Y-1.793 C36.0	N3470 G01 X10.995 Y0.708 C14.0
P1 = P1 + 11	N380 G01 X-0.269 Y-1.844 C37.0	N3480 G01 X11.030 Y0.661 C13.0
	N390 G01 X-0.260 Y-1.894 C38.0	N3490 G01 X11.064 Y0.614 C12.0
G17 G71 G94	N400 G01 X-0.249 Y-1.944 C39.0	N3500 G01 X11.098 Y0.566 C11.0
	N410 G01 X-0.238 Y-1.994 C40.0	N3510 G01 X11.130 Y0.517 C10.0
N10 G01 X-0.000 Y0.000 C0.000 F1000	N420 G01 X-0.226 Y-2.044 C41.0	N3520 G01 X11.162 Y0.468 C9.00
N20 G01 X-0.023 Y-0.046 C1.000	N430 G01 X-0.213 Y-2.094 C42.0	N3530 G01 X11.193 Y0.418 C8.00
N30 G01 X-0.045 Y-0.092 C2.000	N440 G01 X-0.199 Y-2.143 C43.0	N3540 G01 X11.223 Y0.368 C7.00
N40 G01 X-0.066 Y-0.139 C3.000	N450 G01 X-0.184 Y-2.192 C44.0	N3550 G01 X11.252 Y0.317 C6.00
N50 G01 X-0.086 Y-0.186 C4.000	N460 G01 X-0.169 Y-2.241 C45.0	N3560 G01 X11.280 Y0.265 C5.00
N60 G01 X-0.105 Y-0.233 C5.000	N470 G01 X-0.152 Y-2.289 C46.0	N3570 G01 X11.307 Y0.214 C4.00
N70 G01 X-0.124 Y-0.281 C6.000	N480 G01 X-0.135 Y-2.338 C47.0	N3580 G01 X11.334 Y0.161 C3.00
N80 G01 X-0.142 Y-0.329 C7.000	N490 G01 X-0.117 Y-2.386 C48.0	N3590 G01 X11.359 Y0.109 C2.00
N90 G01 X-0.159 Y-0.378 C8.000	N500 G01 X-0.098 Y-2.433 C49.0	N3600 G01 X11.384 Y0.055 C1.00
N100 G01 X-0.175 Y-0.426 C9.00	N510 G01 X-0.078 Y-2.480 C50.0	N3610 G01 X11.407 Y0.002 C0.00
N110 G01 X-0.190 Y-0.475 C10.0		G158 XP1
N120 G01 X-0.204 Y-0.524 C11.0		$ENDWHILE
N130 G01 X-0.218 Y-0.574 C12.0		G01 Z1 = 1 Z2 = 1 Z3 = 1 F50
N140 G01 X-0.231 Y-0.623 C13.0		G01 X-48 F500
N150 G01 X-0.242 Y-0.673 C14.0		
N160 G01 X-0.253 Y-0.723 C15.0		G54
N170 G01 X-0.263 Y-0.774 C16.0		G158
N180 G01 X-0.273 Y-0.824 C17.0		
N190 G01 X-0.281 Y-0.875 C18.0	N3290 G01 X10.237 Y1.434 C32.0	M30
N200 G01 X-0.288 Y-0.925 C19.0	N3300 G01 X10.285 Y1.400 C31.0	
N210 G01 X-0.295 Y-0.976 C20.0	N3310 G01 X10.332 Y1.366 C30.0	

### Appendix A.2. ISO Programs for the Cutting Paths of Figure 18

#### Appendix A.2.1. ISO Program without Tool Tip Correction

V.A.ORGT[2].X = −51
V.A.ORGT[2].Y = 33
V.A.ORGT[2].Z1 = −140.630
V.A.ORGT[2].Z2 = −140.630
V.A.ORGT[2].Z3 = −140.630
V.A.ORGT[2].C = 80
G55
G01 X0 Y0 Z1 = 0 Z2 = 0 Z3 = 0 C0 F100 (PRIORITY 1 BEGIN)
G17 G71 G94
G02 X0.5 Y0.5 I0.5 J0 C90
G02 X1 Y0 I0 J0.5 C180
G02 X0.5 Y-0.5 I-0.5 J0 C270
G02 X0 Y0 I0 J-0.5 C360
G01 Z1 = 1 Z2 = 1 Z3 = 1 F50
G01 X0 F500
G54
G158
M30

#### Appendix A.2.2. ISO Program without Tool Tip Correction

V.A.ORGT[2].X = −53	N310 G01 X-0.387 Y-0.539 C30.0	N3270 G01 X0.693 Y0.285 C326.0
V.A.ORGT[2].Y = 33	N320 G01 X-0.394 Y-0.560 C31.0	N3280 G01 X0.671 Y0.283 C327.0
V.A.ORGT[2].Z1 = −140.630	N330 G01 X-0.402 Y-0.581 C32.0	N3290 G01 X0.649 Y0.280 C328.0
V.A.ORGT[2].Z2 = −140.630	N340 G01 X-0.409 Y-0.603 C33.0	N3200 G01 X0.627 Y0.277 C329.0
V.A.ORGT[2].Z3 = −140.630	N350 G01 X-0.415 Y-0.624 C34.0	N3310 G01 X0.604 Y0.274 C330.0
V.A.ORGT[2].C = 80	N360 G01 X-0.421 Y-0.646 C35.0	N3320 G01 X0.582 Y0.270 C331.0
	N370 G01 X-0.427 Y-0.667 C36.0	N3330 G01 X0.560 Y0.266 C332.0
G55	N380 G01 X-0.433 Y-0.689 C37.0	N3340 G01 X0.539 Y0.261 C333.0
	N390 G01 X-0.438 Y-0.711 C38.0	N3350 G01 X0.517 Y0.256 C334.0
G01 X0 Y0 Z1 = 0 Z2 = 0 Z3 = 0 C0 F100 (PRIORITY 1 BEGIN)	N400 G01 X-0.443 Y-0.733 C39.0	N3360 G01 X0.495 Y0.251 C335.0
	N410 G01 X-0.447 Y-0.754 C40.0	N3370 G01 X0.473 Y0.245 C336.0
	N420 G01 X-0.451 Y-0.776 C41.0	N3380 G01 X0.452 Y0.239 C337.0
N10 G01 X0.000 Y0.000 C0.000	N430 G01 X-0.454 Y-0.799 C42.0	N3390 G01 X0.430 Y0.232 C338.0
N20 G01 X-0.017 Y-0.014 C1.000	N440 G01 X-0.458 Y-0.821 C43.0	N3400 G01 X0.409 Y0.226 C339.0
N30 G01 X-0.034 Y-0.029 C2.000	N450 G01 X-0.460 Y-0.843 C44.0	N3410 G01 X0.388 Y0.218 C340.0
N40 G01 X-0.051 Y-0.044 C3.000	N460 G01 X-0.463 Y-0.865 C45.0	N3420 G01 X0.367 Y0.211 C341.0
N50 G01 X-0.067 Y-0.059 C4.000	N470 G01 X-0.465 Y-0.887 C46.0	N3430 G01 X0.346 Y0.203 C342.0
N60 G01 X-0.083 Y-0.075 C5.000	N480 G01 X-0.466 Y-0.910 C47.0	N3440 G01 X0.325 Y0.194 C343.0
N70 G01 X-0.099 Y-0.090 C6.000	N490 G01 X-0.468 Y-0.932 C48.0	N3450 G01 X0.305 Y0.186 C344.0
N80 G01 X-0.115 Y-0.106 C7.000	N500 G01 X-0.468 Y-0.954 C49.0	N3460 G01 X0.284 Y0.177 C345.0
N90 G01 X-0.130 Y-0.123 C8.000	N510 G01 X-0.469 Y-0.977 C50.0	N3470 G01 X0.264 Y0.167 C346.0
N100 G01 X-0.145 Y-0.139 C9.00	N520 G01 X-0.469 Y-0.999 C51.0	N3480 G01 X0.244 Y0.157 C347.0
N110 G01 X-0.160 Y-0.156 C10.0	N530 G01 X-0.469 Y-1.022 C52.0	N3490 G01 X0.224 Y0.147 C348.0
N120 G01 X-0.174 Y-0.173 C11.0	N540 G01 X-0.468 Y-1.044 C53.0	N3500 G01 X0.204 Y0.137 C349.0
N130 G01 X-0.188 Y-0.191 C12.0	N550 G01 X-0.467 Y-1.066 C54.0	N3510 G01 X0.184 Y0.126 C350.0
N140 G01 X-0.202 Y-0.208 C13.0	N560 G01 X-0.465 Y-1.089 C55.0	N3520 G01 X0.165 Y0.115 C351.0
N150 G01 X-0.216 Y-0.226 C14.0	N570 G01 X-0.463 Y-1.111 C56.0	N3530 G01 X0.146 Y0.104 C352.0
N160 G01 X-0.229 Y-0.244 C15.0	N580 G01 X-0.461 Y-1.133 C57.0	N3540 G01 X0.127 Y0.092 C353.0
N170 G01 X-0.242 Y-0.262 C16.0	N590 G01 X-0.458 Y-1.155 C58.0	N3550 G01 X0.108 Y0.080 C354.0
N180 G01 X-0.254 Y-0.281 C17.0	N600 G01 X-0.455 Y-1.178 C59.0	N3560 G01 X0.089 Y0.067 C355.0
N190 G01 X-0.266 Y-0.300 C18.0	N610 G01 X-0.452 Y-1.200 C60.0	N3570 G01 X0.071 Y0.054 C356.0
N200 G01 X-0.278 Y-0.319 C19.0	N620 G01 X-0.448 Y-1.222 C61.0	N3580 G01 X0.053 Y0.041 C357.0
N210 G01 X-0.290 Y-0.338 C20.0	N630 G01 X-0.444 Y-1.244 C62.0	N3590 G01 X0.035 Y0.028 C358.0
N220 G01 X-0.301 Y-0.357 C21.0	N640 G01 X-0.439 Y-1.266 C63.0	N3600 G01 X0.017 Y0.014 C359.0
N230 G01 X-0.312 Y-0.377 C22.0	N650 G01 X-0.434 Y-1.287 C64.0	N3610 G01 X0.000 Y0.000 C360.0
N240 G01 X-0.323 Y-0.396 C23.0	N660 G01 X-0.429 Y-1.309 C65.0	
N250 G01 X-0.333 Y-0.416 C24.0		G01 Z1 = 0 Z2 = 0 Z3 = 0 F50
N260 G01 X-0.343 Y-0.436 C25.0		G01 X0 F500
N270 G01 X-0.352 Y-0.457 C26.0		G54
N280 G01 X-0.361 Y-0.477 C27.0		G158
N290 G01 X-0.370 Y-0.498 C28.0		M30
N300 G01 X-0.379 Y-0.518 C29.0		
